# Predictability of Lower Incisor Intrusion with Clear Aligners: A Systematic Review of Efficacy and Influencing Factors

**DOI:** 10.3390/jcm14176339

**Published:** 2025-09-08

**Authors:** David Emilio Fracchia, Denis Bignotti, Stefano Lai, Eric Battista, Alessio Verdecchia, Enrico Spinas

**Affiliations:** 1 Department of Surgical Sciences, Postgraduate School in Orthodontics, University of Cagliari, 09124 Cagliari, Italy; david.fracchia@gmail.com (D.E.F.); denis.bignotti3@gmail.com (D.B.); stefano87lai@gmail.com (S.L.); 2 Private Practice, London SE5 9PU, UK; eric@eb-dental.co.uk; 3 Orthodontics Division, Instituto Asturiano de Odontologia, Universidad de Oviedo, 33006 Oviedo, Spain

**Keywords:** clear aligners, lower incisor intrusion, predictability, orthodontic biomechanics

## Abstract

**Background/Objectives:** This systematic review aimed to evaluate the effectiveness and predictability of lower incisor intrusion with clear aligners in permanent dentition, addressing one of the most challenging aspects of vertical tooth movement control in the mandibular anterior region. **Methods:** A comprehensive literature search was conducted across five databases (PubMed, Scopus, Embase, and Cochrane) according to PRISMA guidelines. Eight clinical studies fulfilled the eligibility criteria. Risk of bias was assessed using ROBINS-I, and certainty of evidence was graded with GRADE. Key outcomes included the amount of achieved versus planned intrusion, predictability, treatment protocols, use of auxiliaries, and patient-related factors such as age and compliance. **Results:** Reported mean intrusion values ranged from 0.4 to 1.5 mm, with predictability between 35% and 65%. The effectiveness of intrusion was influenced by the magnitude of planned movement, auxiliaries (e.g., attachments, elastics), refinement strategies, and patient-specific factors. Substantial heterogeneity was present in measurement methods (CBCT, cephalometry, digital models) and clinical protocols (aligner change intervals, refinement frequency), preventing meta-analysis. Seven of the eight studies were rated as having a serious risk of bias, and the overall certainty of evidence was moderate to low. Long-term outcomes and patient-centered measures were not adequately assessed. **Conclusions:** Within the limitations of the available evidence, lower incisor intrusion with clear aligners may be considered a feasible orthodontic option when supported by biomechanically informed clinical management. However, conclusions should be interpreted with caution due to heterogeneity, high risk of bias, and lack of long-term data. Further standardized studies with longer follow-up are required to strengthen reliability and clinical applicability.

## 1. Introduction

The use of traditional orthodontic devices, such as brackets, ligatures, archwires, and other components, can hinder proper oral hygiene, affect dental esthetics, and cause patient discomfort [1,2,3]. Recent advances in computer-aided design and manufacturing (CAD/CAM) and in dental materials have stimulated increasing demand for plastic systems. As a result, plastic orthodontic solutions specifically developed for adult patients were introduced [4]. The concept of using multiple aligners was first proposed by Kesling in 1945 [5] and later adapted in various forms, such as Ponitz’s removable plastic retainer (Essix^®^) [6], Modlin [7], and McNamara et al. [8]. Invisalign® (Align Technology, San Jose, California), introduced in 1997, transformed Kesling’s concept into a widely used orthodontic solution and marked the beginning of modern aligner therapy [9]. Although high-quality evidence remains limited, aligner companies often make strong claims regarding treatment effectiveness and predictability [10]. In principle, aligner therapy should allow correction of malocclusions with close agreement between digital planning and clinical outcomes. However, even after multiple refinement stages, several types of tooth movement remain unpredictable [11]. Since the first systematic review on clear aligner therapy by Lagravère and Flores-Mir in 2005 [12], numerous studies have evaluated various aspects of aligner therapy [7,8,10,11,12,13,14,15,16], mostly comparing the accuracy of tooth movement and malocclusion correction with fixed appliances [4,10,13,14,15]. A smaller number of reviews have addressed the predictability of specific movements, including canine and molar rotations, incisor intrusion, and molar extrusion [16,17,18,19,20]. These studies suggest that aligners may not always achieve outcomes identical to digital plans. Among these movements, mandibular anterior intrusion represents a particularly complex clinical and biomechanical challenge. Compared with the maxillary anterior region, the mandibular symphysis provides reduced alveolar bone support and limited space for force application, increasing the risk of root resorption and periodontal damage. Furthermore, the smaller crown size and limited surface for attachments complicate anchorage and force control, making the predictability of this movement lower than that of maxillary intrusion. Clinically, the intrusion of mandibular anterior teeth (MAT) is critical for deep overbite correction and Class II treatment [21,22], yet evidence on its effectiveness with clear aligners remains scarce. Recognizing this gap, the present systematic review aimed to investigate the predictability of lower incisor intrusion with aligners and the main factors influencing its outcomes.

## 2. Materials and Methods

The current systematic review was conducted in adherence to the PRISMA guidelines, which outline the Preferred Reporting Items for Systematic Reviews and Meta-Analyses [23]. The review protocol was formally registered within PROSPERO, the International Prospective Register of Systematic Reviews, under the registration number CRD420251012973. The primary research question was: *“In patients with permanent dentition, how effective are clear aligners for lower incisor intrusion?”.*

### 2.1. Eligibility Criteria

The inclusion and exclusion criteria for this systematic review were established in accordance with the PICO framework (Population, Intervention, Comparison, Outcome). This framework was applied to ensure a comprehensive and precise selection, facilitating the identification of relevant studies while minimizing the risk of bias. The criteria were defined as follows: “(P) patients with permanent dentition undergoing orthodontic treatment with clear aligners. (I) Use of clear aligners for the intrusion of lower incisors. (C) Studies analyzing the effectiveness of clear aligners, with or without comparison to other orthodontic methodologies. (O) Quantification of achieved intrusion and treatment duration assessed using CBCT, orthodontic setup software, or digital models.

The studies included were retrospective and prospective clinical trials (R&PCTs).

Exclusion criteria were as follows:

- Studies involving patients with mixed or deciduous dentition.
- Studies that did not directly analyze lower incisor intrusion.
- Studies lacking quantitative data.
- Studies involving orthodontic treatments with extractions.

Inclusion and exclusion criteria are also summarized in Table 1.

### 2.2. Information Sources and Search Strategy

A thorough search strategy was developed to ensure the comprehensive retrieval of relevant studies. The following online databases were systematically explored: PubMed, Scopus, Embase, Cochrane Central Register of Controlled Trials (CENTRAL), Web of Science and OpenGrey. The search process was carried out up to 20 April 2025, without imposing any restrictions related to publication date or language, thereby maximizing the inclusivity of the results. For each database, the same advanced search approach was consistently applied, utilizing a combination of keywords, Medical Subject Headings (MeSH) terms, and Boolean operators to enhance search precision and completeness. The specific search strategies tailored to each database are comprehensively detailed in Table 2.

### 2.3. Selection Process

The research process was independently conducted by two authors (D.F. and D.B.), who also performed the subsequent screening of the results obtained. To assess the degree of inter-reviewer agreement, Cohen’s kappa coefficient was calculated. The analysis revealed a substantial agreement between the two primary reviewers (κ = 0.66). This value reflected the complexity of applying the inclusion and exclusion criteria, particularly in borderline cases such as unclear reporting of intrusion or mixed outcomes. Any disagreements were resolved through consensus, and when necessary, with the involvement of a third reviewer (S.L.). The initial screening was performed by evaluating the titles and abstracts of the retrieved studies, selecting those deemed potentially eligible, specifically those focusing on tooth movement and clear aligners. Following this preliminary phase, a second screening was conducted by thoroughly reviewing the full texts of the selected articles, in accordance with the predetermined inclusion and exclusion criteria.

### 2.4. Data Collection

The data of interest were selected and extracted independently by the two primary reviewers, following the data extraction model established by the Cochrane Consumers and Communication Review Group. The extracted data were systematically organized into two distinct categories: primary data and specific data. The data were subsequently exported as follows:

Primary Data:

*(a)*  *Author and Year of Publication*
*(b)*  *Country*
*(c)*  *Study Design*
*(d)*  *Number of Patients*
*(e)*  *Mean Age of Patients*
*(f)*  *Gender (M/F)*
*(g)*  *Treatment Duration*
*(h)*  *Main Conclusions*

Specific Data:

*(1)*  *Aligner Brand*
*(2)*  *Intrusion Measurement System*
*(3)*  *Refinement*
*(4)*  *Aligner Change Frequency*
*(5)*  *Predictability of Movement (%)*
*(6)*  *Intrusion Amount (mm)*
*(7)*  *Auxiliary Elements and Attachments*

The primary objectives of this systematic review were to evaluate the effectiveness of clear aligners in achieving lower incisor intrusion in patients with permanent dentition, measuring the amount of intrusion obtained, and assessing the predictability of the movement. Additionally, the review aimed to analyze the variability in effectiveness according to the brand of clear aligners used. The secondary objectives included investigating the influence of clinical and technical factors on the effectiveness of intrusion, such as the frequency of aligner changes, the presence of auxiliary elements and attachments, and the measurement system employed. Moreover, demographic and treatment-related factors, including patient age, gender, and treatment duration, were analyzed to assess their impact on outcomes. Another secondary objective was to evaluate the need for refinement and its effect on the accuracy and amount of intrusion achieved. Finally, the main conclusions from each study were summarized to identify key clinical findings and assess the level of evidence provided. Given the substantial methodological and clinical heterogeneity among the included studies (differences in measurement methods, aligner protocols, refinement strategies, and use of auxiliaries), no meta-analysis was performed. Instead, the results were synthesized narratively. Subgroup analyses were attempted where feasible (e.g., auxiliaries vs. no auxiliaries, measurement method, patient age), but the limited number of studies prevented meaningful quantitative comparisons. Authors were not contacted for missing data, as this information was insufficiently reported in the original studies and could not be reliably retrieved. In particular, variability in measurement methods (CBCT, 3D digital models, cephalometrics) and in clinical protocols (aligner change intervals, refinement strategies, types of attachments) were the main factors that prevented pooling of data into a meta-analysis.

### 2.5. Quality Assessment

Two investigators independently evaluated the risk of bias for each included study by employing the ROBINS-I tool [24], which is specifically designed to assess bias in non-randomized studies. The ROBINS-I tool systematically evaluates the risk of bias across seven key domains, including confounding, selection of participants, classification of interventions, deviations from intended interventions, missing data, measurement of outcomes, and selection of reported results. By critically appraising these domains, ROBINS-I ensures a comprehensive assessment of internal validity. In our review, seven out of eight studies were judged to be at serious risk of bias. The most frequent issues were confounding (limited control for patient-related factors such as age and compliance), measurement bias (differences in assessment methods, e.g., CBCT vs. cephalometry vs. digital models), and misclassification of interventions (incomplete or unclear reporting of aligner protocols and refinements). These methodological limitations reduce the reliability of the findings and require cautious interpretation of the conclusions. To further ensure the robustness and credibility of the findings, the quality and certainty of the evidence for each outcome were systematically assessed using the GRADE approach (Grading of Recommendations, Assessment, Development, and Evaluation) [25]. The GRADE approach provides a transparent and structured method for rating the quality of evidence and the strength of clinical recommendations, categorizing the evidence into four levels (high, moderate, low, very low) based on factors such as study design, consistency of results, directness of evidence, precision, and publication bias. The ROBINS-I tool for risk of bias and the GRADE approach for certainty of evidence were applied independently by two reviewers. Any disagreements were discussed, and consensus was reached with the involvement of a third author (PhD), who has expertise in the field, ensuring a rigorous and transparent evaluation process.

## 3. Results

### 3.1. Study Selection

**Figure 1:**
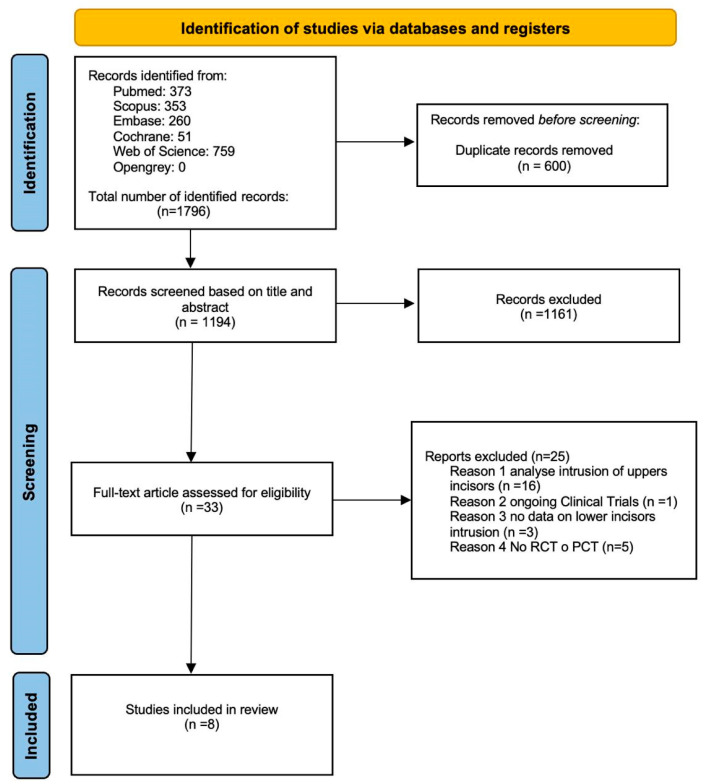
PRISMA flowchart, flow diagram of the performed search.

The database search retrieved 1796 abstracts: 373 from PubMed, 353 from Scopus, 260 from Embase, 51 from Cochrane, 759 from Web of Science, and none from OpenGrey. Following the removal of 600 duplicate records, 1194 papers were screened by evaluating the title and abstract, and subsequently excluded according to the inclusion and exclusion criteria. After this initial screening, 88 studies were assessed for eligibility based on the full-text reading. However, 55 of these articles were not retrieved due to accessibility issues. Consequently, 33 full-text articles were examined for eligibility. After thorough assessment, 25 articles were excluded for the following reasons: 16 articles analyzed the intrusion of upper incisors, one article was related to ongoing clinical trials, three articles lacked data on lower incisor intrusion, and five articles did not involve randomized controlled trials (RCTs) or prospective clinical trials (PCTs). As a result, eight studies were finally included in the qualitative synthesis. The flowchart of the screening process, according to the PRISMA statement, is shown in Figure 1. 

### 3.2. Study Characteristics

The main characteristics of the included studies are reported in Table 3. All included studies are retrospective and prospective clinical trials (RCTs and PCTs) that evaluate the effectiveness of lower incisor intrusion through clear aligners, all performed with Invisalign [26,27,28,29,30,31,32,33]. The studies were conducted in various countries, including China [26], Italy [27,28], Japan [29], United States [30,31,33], and the United Arab Emirates [32]. The studies included in the systematic review were published between 2021 and 2024.

### 3.3. Primary Outcomes

#### 3.3.1. Predictability of Movement (%)

The predictability of lower incisor intrusion movement shows considerable variability among different studies. Bilello et al. [27] reported highly promising outcomes, with predictability rates of 92% for lateral incisor intrusion and 98% for central incisors. In contrast, Al-balaa et al. [26] reported significantly lower predictability at 44.71%. A more complex scenario emerges from the study by Kang et al. [30], where predictability was observed to decrease between aligner sets: in the first cycle, predictability was 42.13% for central incisors and 38.72% for lateral incisors, while in the second cycle, it dropped to 26.89% for central incisors and 25.49% for lateral incisors. Shahabuddin et al. [33] reported an overall predictability of 42.5%. In contrast, Kravitz et al. [31] demonstrated differences between adolescents and adults, with values of 63.5% and 45.3%, respectively. Finally, Fiorillo et al. [28] reported a predictability rate of 47.7% (Table 4).

#### 3.3.2. Intrusion Amount (mm)

The amount of intrusion is a fundamental parameter to assess the effectiveness of the treatment. Fujiyama et al. [29] reported a maximum intrusion value of 2.0 mm, while Kravitz et al. [31] observed intrusion levels of 1.7 mm in adolescents and 0.9 mm in adults. Al-balaa et al. [26] documented an intrusion value of 0.82 mm, while Shahabuddin et al. [33] reported intrusion amounts of 1.09 mm for central incisors and 1.02 mm for lateral incisors (Table 4).

### 3.4. Secondary Outcomes: Influencing Factors

#### 3.4.1. Intrusion Measurement System

Different studies have employed various systems for measuring intrusion, each with its unique features and characteristics. For instance, Al-balaa et al. [26] utilized Cone-Beam Computed Tomography (CBCT), an advanced technique that provides high-precision three-dimensional images, essential for accurately assessing dental intrusion. Conversely, Bilello et al. [27] employed Rhinoceros^®^ software (Robert McNeel & Associates, Seattle, WA, USA), known for its accuracy in three-dimensional modeling. Fiorillo et al. [28] used Geomagic Control X software (Rock Hill, SC, USA) which is commonly employed for structural variation analysis. In a more traditional approach, Fujiyama et al. [29] relied on cephalometric radiographs to assess intrusion. Kang et al. [30] adopted an open-source approach, utilizing 3D Slicer software via the SlicerCMF project (version 4.11.2), which allows customizable three-dimensional analysis. Similarly, Shahabuddin et al. [33] used an updated version of the same software (version 4.9.0). Additionally, Kravitz et al. [31] employed Compare software (version 8.1; GeoDigm, Falcon Heights, MN, USA), while Sadek et al. [32] used eModel 9.0 software (GeoDigm Corporation, Falcon Heights, MN, USA).

#### 3.4.2. Refinement

A crucial aspect in managing orthodontic treatment is the use of refinement, which involves adjustments to the therapeutic plan during treatment. The application of refinement was reported in some studies, such as Bilello et al. [27] and Kang et al. [30], where it was emphasized as an essential phase to improve intrusion accuracy. Conversely, refinement was not reported in the studies by Al-balaa et al. [26], Fiorillo et al. [28], Kravitz et al. [31], Sadek et al. [32], and Shahabuddin et al. [33], while Fujiyama et al. [29] did not specify whether refinement was utilized.

#### 3.4.3. Aligner Change Frequency

The frequency of aligner replacement is a critical factor in determining the success of orthodontic treatment. The analyzed studies exhibit significant variability in this parameter. Al-balaa et al. [26] and Sadek et al. [32] recommend changing aligners every 14 days, whereas Bilello et al. [27], Kravitz et al. [31], and Kang et al. [30] suggest a weekly change (every 7 days). In some cases, such as those described by Fujiyama et al. [29] and Fiorillo et al. [28], the frequency was not explicitly mentioned. Shahabuddin et al. [33] reported a variable frequency ranging from 7 to 14 days, depending on clinical requirements.

#### 3.4.4. Auxiliary Elements and Attachments

The use of auxiliary elements and attachments varies significantly among different studies. Bilello et al. [27] employed a combination of bite ramps, power ridges, elastics, and optimized root control attachments. Kang et al. [30] and Shahabuddin et al. [33] utilized precision bite ramps and optimized attachments, including pressure areas to enhance stability. Kravitz et al. [31] reported the use of maxillary incisor bite ramps and specific attachments for bite control. Fiorillo et al. [28] preferred standardized horizontal attachments, avoiding bite ramps or elastics, while Fujiyama et al. [29] did not employ any auxiliaries other than elastics. Finally, Sadek et al. [32] reported the use of precision bite ramps and pressure areas, while Al-balaa et al. [26] utilized pressure areas without bite ramps. The specific details of predictability, amount of intrusion, and any auxiliary elements and attachments useful for each study are reported in Table 5.

### 3.5. Risk of Bias Assessment

**Figure A1:**
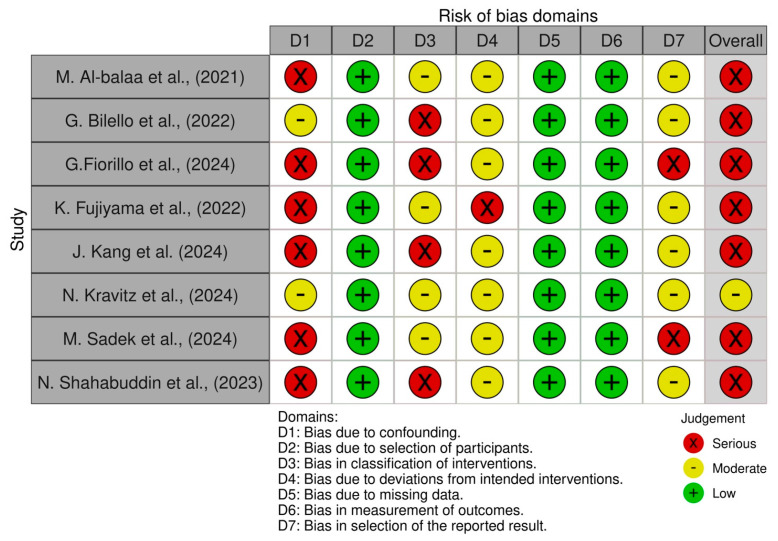
Robins-I Tool [26,27,28,29,30,31,32,33].

According to the ROBINS-I tool (Cochrane Collaboration’s risk of bias assessment tool) [24], the quality of the included studies is summarized in Figure A1. Seven studies were classified as having a serious risk of bias [26,27,28,29,30,32,33], while only one study was judged to be at moderate risk of bias [31]. According to the GRADE approach (Grading of Recommendations, Assessment, Development and Evaluation) [25], the certainty of the evidence is reported in Table A1. Six studies were rated as providing moderate certainty of evidence [26,28,29,30,32,33], and two studies were rated as high certainty [27,31]. The main concerns were confounding, measurement bias, and misclassification of interventions. These limitations directly affect confidence in the outcomes; therefore, the conclusions of this review must be interpreted with caution, avoiding undue generalizations.

## 4. Discussion

The intrusion of mandibular incisors using clear aligners represents one of the most complex and debated movements in contemporary orthodontics. The use of clear aligners is becoming increasingly widespread in daily clinical practice. However, the control of vertical movements remains a significant challenge. Achieving predictable intrusion continues to be difficult [34]. The systematic review conducted reveals a highly heterogeneous body of scientific literature, with reported success rates ranging from 38.72% to 98% [27,30]. This wide variability is not incidental but rather reflects the interaction of multiple biomechanical, methodological, and clinical factors influencing treatment outcomes. The lowest predictability rate (38.72% for the lateral lower incisors) reported by Kang et al. [30] suggests that even in the presence of attachments or bite ramps, aligners alone may not be sufficient to generate controlled intrusive forces, indicating that these auxiliaries do not necessarily enhance the effectiveness of the movement. In contrast, Bilello et al. [27], who employed bite ramps, power ridges, and elastics, achieved significantly higher results, with predictability rates of 92% for lateral incisors and 98% for central incisors. The discrepancy in outcomes between the studies by Bilello et al. [27] and Kang et al. [30] regarding lower incisor intrusion can be attributed to methodological, clinical, and procedural differences. Bilello [27] used occlusal plane superimposition and considered refinements as an integral part of treatment, whereas Kang [30] evaluated each aligner set separately, observing a progressive decline in effectiveness. Moreover, Bilello [27] limited intrusion to ≤2 mm, within a more predictable range, and systematically employed auxiliaries such as bite ramps and elastics. In contrast, Kang’s study [30] reported a possible posterior “bite block” effect hindering anterior intrusion. Lastly, the prospective design and smaller, more controlled sample in Bilello’s study [27] may have contributed to higher predictability. Therefore, predictability values reported in the literature should be interpreted with caution, considering the specific clinical protocols and evaluation criteria employed in each study. Even when biomechanical auxiliaries are properly implemented, the effectiveness of incisor intrusion appears to be constrained by biological and mechanical thresholds. Fiorillo et al. [28] reported a decline in accuracy as the magnitude of the intrusive movement increased, particularly beyond 2 mm. This suggests the existence of a physiological limit, beyond which aligners are no longer capable of maintaining optimal vertical control, increasing the risk of side effects such as tipping or arch bowing. An additional relevant factor highlighted by this review is treatment time, which appears to be a determinant variable in the overall effectiveness of clear aligner therapy. Kang et al. [30] observed a progressive reduction in tooth movement predictability during the later stages of treatment, especially during refinement phases, and additional aligner sets following the initial plan. The data show a decrease in central incisor predictability from an initial 42.13% to a final 26.89%. This downward trend may be attributed to biological adaptations of periodontal tissues or a progressive decline in the mechanical efficiency of the aligners. If confirmed, this would suggest that treatment effectiveness is not constant throughout therapy and may vary based on treatment duration and biomechanical complexity. Clinically, these findings emphasize the importance of continuous and meticulous monitoring throughout all stages of treatment with clear aligners, particularly in identifying signs of delayed or ineffective tooth movements. Such signs may necessitate refinement phases and possible adjustments to biomechanical parameters, including staging recalibration, attachment redesign, or the use of auxiliaries such as temporary anchorage devices (TADs). A proactive and adaptive approach, grounded in continuous assessment of treatment progress, may be critical to achieving high levels of predictability and clinical efficiency. A recent study by Xiao et al. (2025) [35] evaluated, through CBCT analysis and finite element simulations (FEA), the biomechanical effectiveness of mandibular incisor intrusion using clear aligners, identifying frequent unwanted labial or lingual inclinations related to the initial tooth angulation. The authors proposed new attachment designs to improve biomechanical control: Lingual Fossa Excavating Holes (LFEH) for cases with initial labial inclination, and Root Control Ridges (RCR) for marked initial labial or lingual inclinations. The combination of LFEH and labial RCR proved particularly effective in cases with significant initial lingual inclination. The study concludes that personalized attachment design is essential to achieve true vertical intrusion and to minimize the risk of periodontal complications. Age also appears to exert a significant influence on the predictability of tooth movements with clear aligners, representing a clinically relevant variable. Kravitz et al. [31] reported higher intrusion predictability in adolescents (63.5%) compared to adults (45.3%). This difference may be due to several biological, functional, and behavioral factors. Greater bone plasticity and more active skeletal remodeling in adolescence provide a more favorable biological environment for intrusive movement, coupled with generally higher compliance among younger patients. Functionally, adolescents tend to present with lower occlusal forces and less dental wear than adults, potentially facilitating intrusion. The study also found a weak negative correlation between patient age and movement accuracy, indicating a gradual decline in efficacy with increasing age. These findings suggest that chronological age should be considered a relevant clinical parameter during biomechanical planning, especially in deep bite cases relying heavily on lower incisor intrusion. Similar conclusions were drawn by Sadek et al. [32], who emphasized the strategic role of age in the design of complex tooth movements with aligners. Materials also play a crucial role in the effectiveness of clear aligners. Li et al. [36] highlight that thermoplastic materials such as PETG and TPU are particularly reliable due to their mechanical stability compared to 3D printing materials. This distinction is further supported by the study of Mu et al. [37], which demonstrates how the choice of material significantly influences orthodontic efficiency. Although specific data on intrusion were not provided, results suggest that material type influences force transmission, an aspect particularly relevant for intrusive movements. A meaningful contribution to the understanding of lower incisor intrusion comes from Shahabuddin et al. [33], who reported an average predictability of 42.5% in adult patients treated with clear aligners. Despite the use of bite ramps and inter-arch elastics by some patients, no statistically significant difference in treatment efficacy was observed, suggesting that anchorage and movement sequencing may be more decisive factors. These findings support the notion that treatment of deep bites with aligners typically requires refinement phases and overcorrection strategies during initial planning [38]. A recurring issue among the selected studies is the considerable heterogeneity in measurement methods used to evaluate intrusion. The literature includes CBCT [26], two-dimensional cephalometric radiographs [32], and three-dimensional software such as SlicerCMF or GeoMagic [27]. While all these approaches are potentially valid and complementary, their variability complicates direct result comparison and limits the generalizability of clinical conclusions. Standardizing measurement protocols in future studies could enhance methodological robustness and the reliability of evidence. In addition, the substantial methodological heterogeneity across studies—regarding measurement methods, aligner protocols, refinement strategies, and auxiliaries—precluded both subgroup analyses and quantitative meta-analysis. For this reason, the present synthesis was limited to a descriptive and narrative approach, which should be considered when interpreting the results. The findings of Zhu et al. [39], who employed finite element analysis and demonstrated a consistent tendency toward labial proclination of the lower incisors during Spee curve correction. This supports the notion that vertical control remains limited even when intrusive forces are applied, highlighting the need for more refined biomechanical strategies. Although excluded from this systematic review due to selection criteria, several finite element modeling studies provide meaningful insights into the biomechanical implications of orthodontic movements. Zhu et al. [40], for instance, described the “bowing effect” during en-masse retraction in an in vitro setting, which may compromise vertical control despite planned intrusion. Similarly, Yixin Li et al. [41] noted that excessive labial root inclination, reflected by increased IMPA angle, may elevate the risk of bone fenestration. These findings underscore the importance of custom aligner design to mitigate biomechanical side effects and enhance treatment predictability. Recent FEM-based studies have shown that TAD positioning significantly influences force distribution, intrusion patterns, and side effects such as molar extrusion [42]. Xiao et al. [43] confirmed that force vector and insertion site affect labial inclination of incisors and, in some cases, canine extrusion, as well as PDL stress distribution, potentially increasing root resorption risk. These findings, though focused on the maxilla, are relevant for planning safer and more predictable lower incisor intrusion. A systematic review reported that maxillary TADs yield greater and more selective intrusion with less molar extrusion than conventional arches [44], suggesting potential benefits for skeletal anchorage in the mandible. However, evidence quality is low, and long-term outcomes remain unclear. Regarding biological safety, orthodontically induced root resorption during intrusion averages 0.72 mm and is generally considered clinically acceptable, though data largely derive from fixed appliances and favorable root morphology [45]. In contrast, the single-rooted anatomy and variable morphology of lower incisors may predispose them to greater resorption risk, particularly under poorly controlled intrusive forces. Evidence on the biological impact of aligners in this specific movement remains sparse and fragmented. Comparative studies, such as those by Xia et al. [46] and Mario et al. [47], have shown that fixed appliances may outperform aligners in anterior intrusion, with aligners more likely to produce extrusion effects and show reduced stability in complex movements. Attachments also play a critical role in lower incisor intrusion. The systematic review by Nucera et al. [48] analyzed the impact of attachments on various tooth movements, with mixed results regarding intrusion. Some studies reported enhanced intrusion effectiveness with vestibular attachments due to improved aligner retention and force delivery. However, specific evidence on lower anterior teeth remains lacking, and results across different attachment shapes and placements remain inconclusive. Thus, case-specific attachment design and precise staging remain fundamental. Patient compliance should be considered a key factor influencing the outcomes of clear aligner therapy. This aspect becomes particularly critical in movements that are inherently challenging with aligners, such as lower incisor intrusion. Insufficient wear time may compromise the biomechanical effectiveness of the appliance, lower the predictability of results, and potentially increase unwanted side effects. Emphasizing patient adherence is therefore essential to ensure reliable clinical performance.

Finite element analyses and in vitro biomechanical studies, although excluded from our systematic review, provide important insights into the mechanisms underlying intrusion failures. Zhu et al. [39] demonstrated that deep bite correction protocols combining distalization and posterior extrusion resulted in uncontrolled proclination of mandibular incisors and increased periodontal ligament stress. Zhu et al. [40] confirmed that even when intrusive activation is added, the “bowing effect” persists, limiting vertical control of the incisors. Liu and Hu [49] reported that different intrusion strategies distribute forces unevenly across anterior and posterior teeth, with premolars frequently subjected to extrusive forces. Li et al. [41] showed that aligner deformation consistently produced labial tipping moments on mandibular incisors despite intrusive activation. Together, these biomechanical data explain why mandibular incisor intrusion with aligners shows a modest magnitude and high variability in clinical studies. This review presents several limitations. The number of eligible studies was limited, with generally small sample sizes, which reduces statistical power and increases the potential risk of bias. Substantial methodological and clinical heterogeneity was observed in study design, sample characteristics, aligner protocols, use of auxiliaries, and measurement methods, preventing a reliable meta-analysis and restricting the synthesis to a descriptive level. Most studies were assessed as being at serious risk of bias according to ROBINS-I, and few reported measures of dispersion, thereby limiting the precision of outcomes. In addition, the lack of long-term follow-up data leaves the stability of mandibular incisor intrusion with aligners uncertain, and patient-reported outcomes were not systematically evaluated. Taken together, these factors reduce the overall strength and applicability of the conclusions.

Nevertheless, current evidence indicates that mandibular incisor intrusion with clear aligners is achievable, although the magnitude and predictability remain limited. More robust evidence from larger, well-designed prospective trials with standardized protocols, post-treatment follow-up, and systematic inclusion of patient-reported outcomes is needed to draw more definitive conclusions.

From a clinical perspective, the present findings indicate that mandibular incisor intrusion with clear aligners is achievable but of limited magnitude (≈0.8–1.7 mm) and modest predictability (≈40–64%). Clinicians should, therefore, anticipate the need for refinements and consider the systematic use of auxiliaries such as bite ramps and optimized attachments to enhance vertical control. Overcorrection may be required in the digital setup, particularly in adult patients, who consistently show lower predictability than adolescents. Close monitoring of periodontal health is also recommended, since aligner-related force systems may produce labial tipping stresses on the incisors. Overall, treatment planning for deep bite correction with aligners should balance expectations of limited intrusion with the strategic use of auxiliaries and refinements.

## 5. Conclusions

This systematic review suggests that lower incisor intrusion with clear aligners may be clinically feasible, though predictability varies according to several clinical parameters. Intrusions of approximately 1.5–2.0 mm appear to show higher accuracy (60–98%) when auxiliaries are used appropriately and staging is carefully managed, whereas planned intrusions beyond 2.0 mm show reduced predictability, often below 50%, requiring refinements or overcorrection strategies.

These findings must be interpreted with caution due to substantial methodological heterogeneity, variability in measurement techniques, and the lack of long-term follow-up, which prevent definitive conclusions and limit generalizability. Biological factors such as bone density and periodontal response, although potentially relevant, remain insufficiently explored.

Future studies should prioritize standardized protocols, refined biomechanical strategies, longitudinal assessments, and patient-reported outcomes. Until stronger evidence becomes available, clinicians are advised to adopt individualized treatment approaches, integrating auxiliaries as indicated and planning for possible refinements, particularly in adult patients or in cases requiring greater degrees of intrusion.

## Figures and Tables

**Table 1:** PICO framework and Inclusion and exclusion criteria.

PICOS	Inclusion Criteria	Exclusion Criteria
Participant	Adult and adolescent patients with permanent dentition	Patients with mixed or deciduous dentition
Intervention	Use of clear aligners for the intrusion of lower incisors without extractions	Treatments with clear aligners without lower incisor intrusion, treatments with extractions.
Comparison	Studies analyzing the effectiveness of clear aligners in lower incisors intrusion, with or without comparison to other orthodontic methodologies	Studies that do not include any analysis of the effectiveness of lower incisor intrusion with clear aligners.
Outcome	Effectiveness of lower incisor intrusion measured through CBCT, setup software, or cephalometric analysis.	Studies without measurable quantitative data.
Study design	R&PCTs	Case–control, cohort, cross-sectional, Nr-RCTs 2; reviews; case reports; case series; in vitro; and animal studies

**Table 2:** Search strategy for each database.

Database	Search Strategy
PubMed	(“Tooth Movement Techniques”[MeSH Terms] OR “Orthodontic Anchorage Procedures”[MeSH Terms]) AND (“Clear Aligners”[All Fields] OR “Invisalign”[All Fields])
Scopus	(TITLE-ABS-KEY (“tooth movement techniques” OR “orthodontic anchorage procedures”) AND TITLE-ABS-KEY (“clear aligners” OR “Invisalign” OR “removable aligners”))
Embase	(‘Tooth movement’/exp OR ‘orthodontic anchorage’/exp) AND (‘clear aligner’/exp OR ‘invisalign’ OR ‘removable orthodontic appliance’)
Cochrane Central Register of Controlled Trials	(“Tooth movement” OR “orthodontic anchorage”) AND (“clear aligner” OR “Invisalign” OR “removable orthodontic appliance”)
Web of Science	(((TS = (“Tooth Movement Techniques”)) OR TS = (“Orthodontic Anchorage Procedures”)) AND TS = (“Clear Aligners”)) OR TS = (“Invisalign”)

Detailed descriptions are given of the search conducted in the five selected databases: PubMed, Scopus, Web of Science, Embase, and the Cochrane Central Register of Controlled Trials (CENTRAL). The search was customized to each database.

**Table 3:** Primary Data, Characteristics of Included Studies.

Author (Year)	Country	Study Design	Number of Patients	Mean Age (Years)	Gender (M/F)	Treatment Duration (Months)	Main Conclusions
Al-balaa et al. (2021) [26]	China	RCT	22	23.74	10/12	Average 19.27	The mandibular incisors exhibited the least precise tooth movement, with an intrusion predictability of 44.71%.
Bilello et al. (2022) [27]	Italy	PCT	10	34.8 ± 14	3/7	Not specified	The mandibular incisors demonstrated a high predictability of intrusion (92.2%), but accuracy decreased for movements exceeding 2 mm.
Fiorillo et al. (2024) [28]	Italy	RCT	25	32.28	12/13	Not specified	Larger vertical changes were correlated with larger errors.
Fujiyama et al. (2022) [29]	Japan	RCT	25 Invisalign 23 fixed appliances	23.3 ± 8.5	7/18	31.9 ± 8.6	Deep overbite correction was primarily achieved through 2.0 mm mandibular incisor intrusion, along with 1.0 mm maxillary incisor intrusion. Treatment duration was comparable to fixed appliances.
Kang et al. (2024) [30]	USA	RCT	20	32.63 ± 11.88	7/13	22.96 ± 12.34	The vertical movement of lower incisors has moderate predictability in the first set of aligners but decreases in refinements.
Kravitz et al. (2024) [31]	USA	PCT	58 (29 adolescents, 29 adults)	-Adolescents: 15.1 -Adults: 40.7	16/42	Not specified	Mandibular incisor intrusion was more accurate in adolescents (63.5%) than adults (45.3%), with greater intrusion (1.7 mm vs. 0.9 mm). Accuracy decreased with age.
Sadek et al. (2024) [32]	UAE	RCT	34	30.65 ± 11.80	Not specified	16.14 ± 6.42	Lower incisor intrusion with aligners shows limited predictability, with unplanned vertical movements being more pronounced in anterior teeth compared to posterior teeth.
Shahabuddin et al. (2023) [33]	USA	RCT	24	32.8 ± 11.9	10/14	11.04 ± 4.14	Intrusion success increased slightly with bite ramps and elastics, but not significantly. Planned vs. achieved movements showed significant discrepancies. Overcorrection and refinements are needed.

**Table 4:** Quantitative outcomes of mandibular incisor intrusion with clear aligners.

Study	Planned (mm)	Achieved (mm)	Predictability (%)
Al-balaa et al. (2021) [26]	1.8	0.82	51.2
Bilello et al. (2022) [27]	≤2.0	NR	92
Fiorillo et al. (2024) [28]	NR	1.15–2.11 diff.	41–51
Fujiyama et al. (2022) [29]	NR	~2.0	NR
Kang et al. (2024) [30]	NR	1.48–1.59	27–42
Kravitz et al. (2024) [31]	NR	0.9–1.7	45–64
Sadek et al. (2024) [32]	NR	NR	NR
Shahabuddin et al. (2023) [33]	NR	1.02–1.09	42.5

Abbreviations: NR = not reported.

**Table 5:** Secondary parameters reported in included studies on lower incisor intrusion with clear aligners.

Author (Year)	Aligner Brand	Intrusion Measurement System	Refinement	Aligner Change Frequency	Auxiliary Elements and Attachments
Al-balaa et al. (2021) [26]	Invisalign	CBCT	No	14 days	Pressure areas, no bite ramps
Bilello et al. (2022) [27]	Invisalign	Rhinoceros® software (Robert McNeel & Associates, USA)	Yes	7 days	Bite ramps, power ridges, elastics, optimized root control attachments
Fiorillo et al. (2024) [28]	Invisalign	Geomagic Control X software (Rock Hill, SC, USA).	No	Not specified	Standardized horizontal attachments on premolars and molars, no bite ramps or elastics used
Fujiyama et al. (2022) [29]	Invisalign	Cephalometric radiographs	Not specified	Not specified	No auxiliary appliances other than elastics were used
Kang et al. (2024) [30]	Invisalign	3D Slicer software via the SlicerCMF project (cmf.slicer.org, open-source, version 4.11.2).	Yes	7–14 days	Bite ramps, optimized rotation attachments, optimized deep bite attachments, and conventional rectangular attachments
Kravitz et al. (2024) [31]	Invisalign Teen (Adolescents), Invisalign Full (Adults)	Compare (version 8.1; GeoDigm, Falcon Heights, Minn)	No	7 days	Maxillary incisor bite ramps, G5 attachments on mandibular premolars and first molars, 4.0 mm beveled attachments on mandibular lateral incisors and canines. Mandibular premolars and first molars extruded 0.5 mm, canines and incisors intruded progressively. Final: 0.0 mm overbite, heavy posterior contacts, no interproximal reduction.
Sadek et al. (2024) [32]	Invisalign	eModel 9.0 software (GeoDigm Corporation, Falcon Heights, MN)	Not specified	14 days	Optimized deep bite attachments, precision bite ramps, pressure areas
Shahabuddin et al. (2023) [33]	Invisalign	3D Slicer via the SlicerCMF project (version 4.9.0; cmf.slicer.org))	No	7–14 days	Precision bite ramps, optimized deepbite attachments, new pressure areas on lingual surfaces

## Data Availability

All data supporting the findings of this study are contained within the article. No additional data are available.

## Appendix A

**Table A1:** GRADE Assessment of Certainty in Included Studies.

Certainty of Assessment						Certainty
Study	Risk of Bias	Inconsistency	Indirectness	Imprecision	Other Considerations	
M. Al-balaa et al. (2021) [26]	Moderate	Low	Moderate	Moderate	None	Moderate
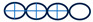
G. Bilello et al. (2022) [27]	Low	Low	Low	Low	None	High
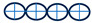
G. Fiorillo et al. (2024) [28]	Moderate	Moderate	Moderate	Moderate	None	Moderate
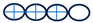
K. Fujiyama et al. (2022) [29]	Moderate	Low	Moderate	Moderate	None	Moderate
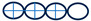
J. Kang et al. (2024) [30]	Moderate	Moderate	Moderate	Moderate	None	Moderate
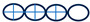
N. Kravitz et al. (2024) [31]	Low	Low	Low	Low	None	High
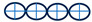
M. Sadek et al. (2024) [32]	Moderate	Moderate	Moderate	Moderate	None	Moderate
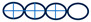
N. Shahabuddin et al. (2023) [33]	Moderate	Moderate	Moderate	Moderate	None	Moderate
